# Risk Factors of Progression to Active Tuberculosis in Rheumatic Patients With Latent Tuberculosis: A Retrospective Study

**DOI:** 10.1002/iid3.70270

**Published:** 2025-10-15

**Authors:** Fengjuan Wang, Lei Zhou, Xiaoyan Hao, Jiayun Liu

**Affiliations:** ^1^ Department of Clinical Laboratory Medicine, Xijing Hospital Fourth Military Medical University Xi′an China

**Keywords:** active tuberculosis, latent tuberculosis infection, rheumatic diseases, risk factor

## Abstract

**Background:**

In rheumatism patients, the immune system erroneously attacks the body′s own tissues. This impairs the body′s defense against external pathogens and is a contributing factor to the occurrence of tuberculosis infection. The primary objective of this investigation was to examine the risk factors for the progression from latent tuberculosis infection (LTBI) to active tuberculosis (ATB) in patients with rheumatic diseases (RD).

**Methods:**

RD cover a wide range of disorders affecting the skeletal system, joints, and adjacent soft tissues. When the human body is infected by *Mycobacterium tuberculosis*, the condition is classified as either LTBI or ATB, depending on the presence or absence of typical clinical symptoms. A retrospective study was conducted at the Xijing Hospital of the Fourth Military Medical University. Specifically, the Laboratory Information System was used to investigate patients diagnosed with RD from January 2012 to October 2022.

**Results:**

The study included a total of 32,235 individuals diagnosed with rheumatism, of whom only 18.60% were screened for LTBI. The overall incidence of LTBI was 25.33%. Among the 629 RD inpatients with LTBI, systemic lupus erythematosus (SLE) and rheumatoid arthritis (RA) accounted for half, and 56.44% received glucocorticoid (GC) therapy. Risk‐factor assessment for ATB was conducted in 247 cases. A GC dose of 20 mg/day or more was an independent risk factor for LTBI activation (odds ratio = 3.59, 95% CI: 1.26–10.29, *p* = 0.017).

**Conclusion:**

In China, RD patients have a relatively high risk of LTBI. In clinical practice, LTBI screening should be routinely performed for RD patients before initiating GC therapy at a dose of ≥ 20 mg/day. For patients with SLE and RA undergoing continuous GC treatment, close monitoring is essential. In addition, clinicians should enhance the diagnostic pathways and treatment management for these patients to prevent the occurrence of ATB.

## 1 Introduction

The World Health Organization [1, 2] estimates that China has the third‐highest tuberculosis (TB) burden in the world, approximately 2–3 billion individuals worldwide are latently infected with *Mycobacterium tuberculosis* (*Mtb*), and 5%–10% of these individuals will develop active symptoms from their TB infection during their lifetime [3]. The reactivation of latent tuberculosis infection (LTBI) plays a significant role in the overall incidence of active tuberculosis (ATB), particularly among high‐risk populations. It has been estimated that China has approximately 20 million individuals with rheumatic diseases [4]. However, there is a paucity of comprehensive epidemiological data pertaining to the prevalence of LTBI among individuals with rheumatic conditions. The use of biologic agents in China has given rise to considerable concern. Consequently, it is of the utmost importance to conduct LTBI screening among immunosuppressed patients, as there is a significant risk of progression to ATB [5]. The incidence of reactivation of LTBI is significantly elevated among individuals receiving biological and immunosuppressive treatment [6]. As of now, there is no gold standard for diagnosing LTBI that is widely recognized. In accordance with the current guidelines in the United States, interferon‐gamma release assay (IGRA) is recommended to diagnose LTBI in adults and older children [7]. In terms of sensitivity, the pooled T‐SPOT.TB assay has been found to outperform both the QuantiFERON assay and the tuberculin skin test (TST). However, it is important to note that in people who have been vaccinated with Bacillus Calmette–Guérin (BCG), TST may show poor specificity, and it may also have reduced sensitivity in individuals with compromised immune systems [8].

The presence of TB infection can introduce complexities in the diagnosis and treatment of individuals with rheumatic conditions. This is particularly significant as rheumatic patients often experience compromised immune systems, resulting in prolonged infection duration and increased mortality rates [9]. Consequently, it becomes imperative to prioritize the prevention of new *Mtb* infections and the progression to ATB in the context of rheumatic patients. The prolonged administration of high‐dose glucocorticoids (GC), immunosuppressants (leflunomide, methotrexate, cyclosporine A) [10, 11], and antitumor necrosis factor (anti‐TNF) biologics has been demonstrated to substantially elevate the likelihood of TB reactivation in individuals with LTBI who suffer from rheumatic conditions [12, 13, 14]. We conducted a comprehensive analysis of the incidence of LTBI among rheumatic inpatients, as well as the associated risk factors contributing to the progression of ATB.

## 2 Methods

### 2.1 Patients and Data Collection

**Figure 1:**
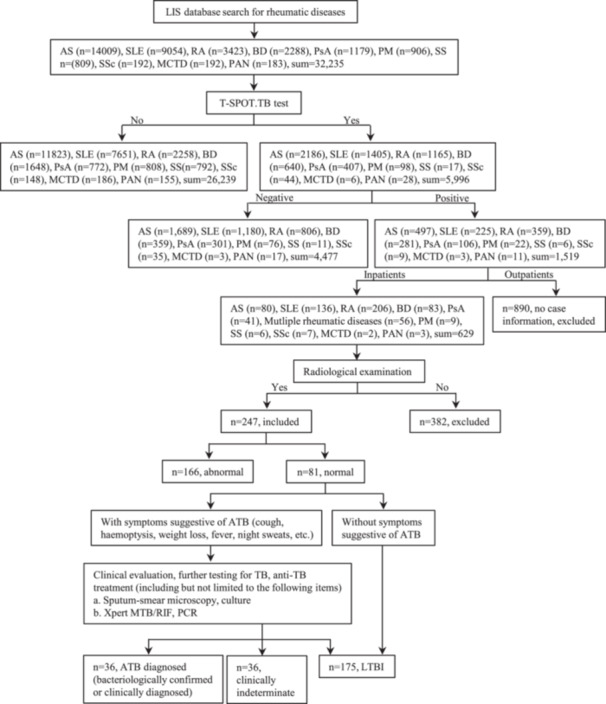
Screening process for ATB. AS, ankylosing spondylitis; ATB, active tuberculosis; BD, Behcet′s disease; EGPA, eosinophilic granulomatosis with polyangiitis; GCA, giant cell arthritis; GPA, granulomatosis with poly‐angiitis; LIS, Laboratory Information System; MCTD, mixed connective tissue disease; MPA, microscopic polyarteritis; PAN, polyarteritis nodosa; PM, polymyositis; PsA, psoriatic arthritis; RA, rheumatoid arthritis; SLE, systemic lupus erythematosus; SS, Sjogren′s syndrome; SSc, systemic sclerosis; TA, Takayasu arthritis; TB, tuberculosis.

This retrospective cohort study was conducted in a country with a high TB burden. As shown in Figure 1, the Laboratory Information System (LIS) was utilized to search for patients with RD who were admitted to the Department of Rheumatology at Xijing Hospital of the Fourth Military Medical University between January 2012 and October 2022. Subsequently, patients who underwent the T‐SPOT.TB assay (Oxford Immunotec Ltd.) was screened to calculate the incidence of LTBI. Finally, the medical records of inpatients and detailed examination items were retrieved from the Hospital Information System for the risk assessment of ATB. The T‐SPOT.TB assay is based on the principle of using the enzyme‐linked immunospot methodology to quantify *Mtb‐*sensitized T cells. According to the manufacturer, the cutoff of the T‐SPOT.TB test is interpreted as follows: if the spot count in each panel minus that in the nil control well is six or greater, the test result is considered positive [15]. A meta‐analysis of 13 studies, which included 726 participants, revealed that the sensitivity of T‐SPOT.TB was 90% (CI, 86%–93%). Moreover, an analysis of six studies involving 290 participants indicated that the specificity of T‐SPOT.TB was 93% (CI, 86%–100%) [16]. The evaluation of T‐SPOT.TB in patients with LTBI demonstrated a high level of sensitivity and was not influenced by immunosuppressive therapy. The positive predictive value of T‐SPOT.TB was 50%, and the negative predictive value ranged from 80% to 97%, suggesting a relatively good negative‐exclusion effect [17, 18, 19, 20, 21]. The inclusion criteria included individuals aged 16 years or older who had been confirmed to have various RD. The exclusion criteria encompassed pregnant individuals and patients with ATB.

### 2.2 Ethical Consideration

The Institutional Ethics Committee of Xijing Hospital of the Fourth Military Medical University thoroughly reviewed and approved all pertinent components of the study, encompassing patients′ clinical data and the accessibility to said data. Given that the study relied on previously examined laboratory data, the requirement for patients′ informed consent was deemed unnecessary.

### 2.3 Case and Control Selection

All inpatients with a positive T‐SPOT.TB results were strictly screened and grouped. The screening process for ATB is summarized in Figure 1.

### 2.4 Definitions

In our study, we defined “T‐SPOT.TB positive” as LTBI individuals, considering the widespread BCG vaccination in China.

The findings of the radiological examinations were analyzed by Lei Zhou, a radiologist with extensive expertise. The findings indicative of LTBI included pleural thickening in the apical or basal regions, fibrotic scarring, densities in the apical area, calcified nodules, and lymphadenopathy in the mediastinal or hilar regions [14].

The calculation of dose equivalents for prednisone was performed based on the following potency ratios: 1 mg prednisone = 0.15 mg betamethasone = 0.15 mg dexamethasone = 0.8 mg triamcinolone = 0.8 mg methylprednisolone = 1 mg prednisolone = 4 mg hydrocortisone = 5 mg cortisone [22]. The diagnostic criteria for ATB can be found in Table 1.

**Table 1:** Diagnostic criteria for ATB.

Diagnostic category	Criteria
Confirmed TB	Culture or Xpert MTB/RIF assay was positive, and (or) typical pathological changes, such as caseous necrosis, granulomatous, etc.
Clinically diagnosed TB	A variety of typical symptoms, such as fever, cough, chest pain, night sweats, weight loss and etc. Laboratory and imaging findings were very supportive of *Mtb*, and anti‐TB therapy was effective.
Clinically uncertain tuberculosis	It was not possible to definitively rule out or confirm a final diagnosis of tuberculosis.

Abbreviations: ATB, active tuberculosis; *Mtb*: *Mycobacterium tuberculosis*; TB, tuberculosis.

### 2.5 Statistical Analysis

The Kolmogorov‐Smirnov test was used to evaluate the normality of numerical variables, with mean ± standard deviation and non‐normal variables described using median and interquartile range (IQR). The presentation of categorical variables involved the use of frequencies and proportions. The study used *t*‐test and Mann–Whitney *U* tests for continuous variables and Chi‐squared or Fisher′s exact tests for categorical data, analyzed using the Beckman Coulter DxAI platform (https://www.xsmartanalysis.com/beckman/login/). For every analysis, a *p* < 0.05 was deemed statistically significant. For every odds ratio (OR), we calculated the 95% confidence interval (95% CI). A two‐sided method was used to determine all *p* values.

## 3 Results

### 3.1 Demographic Characteristics

**Figure 2:**
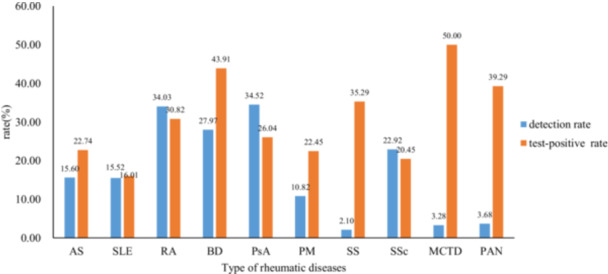
T‐SPOT.TB assay in patients with rheumatic diseases. The blue square represent T‐SPOT. TB test rate, and the orange square represent positive T‐SPOT.TB test rate. AS, ankylosing spondylitis; BD, Behcet′s disease; MCTD, mixed connective tissue disease; PAN, polyarteritis nodosa; PM, polymyositis; PsA, psoriatic arthritis; RA, rheumatoid arthritis; SLE, systemic lupus erythematosus; SS, Sjogren′s syndrome; SSc, systemic sclerosis.

We searched a total of 32,235 patients with rheumatic patients in the Laboratory Information System of Xijing Hospital of the Fourth Military Medical University in the past 10 years, among which 5996 cases (18.60%) were detected using T‐SPOT.TB assay. Overall T‐SPOT.TB positive rate is 25.33% (1519/5996 × 100%), each disease T‐SPOT. TB positive rate from 16.01% to 50.00% (Figure 2). The study included a total of 629 rheumatic inpatients with LTBI, with a median age of 47 years [IQR: 33–59]. Among the participants, 61.37% were female, 5.09% had diabetes, 14.47% had hypertension, 12.72% had respiratory tract infections, and 14.79% had pulmonary infections. Of the total population, 11.13% had a history of tuberculosis, 19.55% had received prophylactic anti‐TB therapy, 56.44% had used GC, 86.17% had used immunosuppressants, and 10.65% had used anti‐TNF biologics. The largest proportion of inpatients belonged to SLE group (136, 21.62%) and RA group (206, 32.75%), accounting for 54.37% of the total population. Table 2 displays the general characteristics of the participants. A comprehensive screening was conducted on 247 inpatients diagnosed with rheumatic conditions with LTBI to identify potential risk factors.

**Table 2:** General characteristics of 629 rheumatic inpatients with LTBI conditions.

Type of rheumatic diseases	AS	SLE	RA	BD	PsA	Mutliple rheumatic diseases	PM	SS	SSc	MCTD	PAN
	*n* = 80	*n* = 136	*n* = 206	*n* = 83	*n* = 41	*n* = 56	*n* = 9	*n* = 6	*n* = 7	*n* = 2	*n* = 3
Male (*n*, %)	62 (77.50)	13 (9.56)	77 (37.38)	41 (49.40)	35 (85.37)	8 (14.29)	2 (22.22)	0	4 (57.14)	0	3 (100.00)
Median age, year	37 [27–50]	40 [29.25–50]	58 [48–65]	35 [28–46]	43 [31.5–52]	55 [44–60]	48 [30–54.50]	46 [32.25–61]	47 [36–56]	45 ± 2.12	21 ± 2.08
Median duration of RD, months	96 [60–168]	60 [9.25–120]	84 [36–168]	60 [12–120]	60 [18–108]	90 [36–177]	12 [2.50–48]	60.5 [40.50–222]	36 [6–120]	50.50 ± 64.34	24 ± 12
Diabetes (*n*, %)	2 (2.50)	2 (1.47)	23 (11.17)	1 (1.20)	2 (4.88)	0	1 (11.11)	0	1 (14.29)	0	0
Hypertensive (*n*, %)	10 (12.50)	30 (22.06)	36 (17.48)	0	6 (14.63)	4 (7.14)	0	2 (33.33)	1 (14.29)	2 (100.00)	0
Respiratory tract infection (*n*, %)	4 (5.00)	28 (20.59)	27 (13.11)	9 (10.84)	1 (2.44)	7 (12.50)	2 (22.22)	1 (16.67)	1 (14.29)	0	0
Pulmonary infection (*n*, %)	4 (5.00)	21 (15.44)	42 (20.39)	5 (6.02)	1 (2.44)	12 (2.14)	5 (55.56)	1 (16.67)	2 (28.57)	0	0
GC (*n*, %)	2 (2.50)	131 (96.32)	86 (41.75)	69 (83.13)	2 (4.88)	45 (80.36)	8 (88.89)	4 (66.67)	6 (85.71)	1 (50.00)	1 (33.33)
Immunosuppressants	61 (76.25)	127 (93.38)	196 (95.15)	57 (68.67)	27 (65.85)	53 (94.64)	7 (77.78)	6 (100.00)	6 (85.71)	2 (100.00)	0
Anti‐TNF therapy (*n*, %)	18 (22.50)	0	23 (11.17)	3 (3.61)	18 (43.90)	5 (8.93)	0	0	0	0	0
Anti‐TB therapy (*n*, %)	21 (26.25)	28 (20.59)	31 (15.05)	24 (28.92)	6 (14.63)	7 (12.50)	3 (33.33)	2 (33.33)	0	1 (50.00)	0
TB history (*n*, %)	9 (11.25)	15 (11.03)	28 (13.59)	6 (7.23)	4 (9.76)	5 (8.93)	1 (11.11)	2 (33.33)	0	0	0

Abbreviations: AS, ankylosing spondylitis; BD, Behcet′sdisease; MCTD, mixed connective tissue diseases; Mutliplerheumatic diseases, at least two types of rheumatic diseases; PAN, polyarteritis nodosa; PM, polymyositis; PsA, psoriatic arthritis; RA, rheumatoid arthritis; SLE, systemic lupus erythematosus; SS, Sjogren′s syndrome; SSc, systemic sclerosis; TB, tuberculosis; TNF, tumor necrosis factor.

### 3.2 Screening Process of ATB

According to the recommendations of the American College of Rheumatology, upon obtaining a positive T‐SPOT.TB result, a chest Computed Tomography (CT)/X‐ray mention radiograph should be carried out first. Subsequently, relevant examinations and screenings for ATB should be conducted [23]. Our survey results show that among the 629 RD inpatients diagnosed with LTBI, only 247 underwent imaging examinations, of whom 72 met the diagnostic criteria for ATB. Among these cases, 28 were confirmed by microbiology, 3 were confirmed by histological examination, 5 were confirmed by clinical diagnosis, and 36 were classified as clinically indeterminate. Within the group of 36 ATB cases, 29 (80.56%) cases had pulmonary TB, 4 (11.11%) cases had meningitis TB, 1 (2.78%) case had joint TB, and 1 (2.78%) case had pancreatic TB.

### 3.3 Analysis of Influencing Factors

In the LTBI group, 81/175 had normal chest CT scans, 11/175 had normal X‐ray and radiograph, 78/175 had acid‐fast staining that was negative, 10/175 had PCR that was negative, and 25/175 had Cepheid GeneXpert MTB/RIF assay that was negative. The study excluded 36 cases of clinically indeterminate ATB, focusing instead on the analysis of 211 cases to identify risk factors associated with the progression of LTBI to ATB. In the ATB group, 29/36 had abnormal chest CT scans, 15/36 had acid‐fast staining that was positive, 12/36 had PCR that was positive, and 8/36 had Cepheid GeneXpert MTB/RIF assay that was positive. The findings of the univariate analysis in Table 3 show that SLE (OR = 4.99, 95% CI: 2.34‐10.66, *p*＜0.001), GC dose (OR = 4.39, 95% CI: 1.79–10.76, *p* = 0.001), and TB history (OR = 4.97 95% CI: 1.88‐13.12, *p* = 0.001) exhibit a significant association with LTBI reactivation. Factors with *p* < 0.05 were included in the binary regression analysis, and only GC ≥ 20 mg/day (OR = 3.59, 95% CI: 1.26–10.29, *p* = 0.017) was an independent risk factor for progression to ATB in RD with LTBI, as shown in Table 4.

**Table 3:** Risk factors of ATB in rheumatic patients with LTBI in univariate analysis.

Variables	ATB *n* = 36	LTBI *n* = 175	OR (95% CI)	*p* value
Male, *n* (%)	10 (27.78)	79 (45.14)	1.00	
Female, *n* (%)	26 (72.22)	96 (54.86)	2.14 (0.97–4.70)	0.058
Age interval in years, *n* (%)				
＜ 40	10 (27.78)	84 (48.00)	1.00	
≥ 40	26 (72.22)	91 (52.00)	0.87 (0.42–1.78)	0.697
Disease duration, *n* (%)				
＜ 48 months	16 (44.44)	72 (41.14)	1.00	
≥ 48 months	20 (55.56)	103 (58.86)	1.14 (0.56–2.36)	0.715
Type of rheumatic diseases				
Other rheumatic diseases, *n* (%)	17 (47.22)	143 (81.71)	1.00	
SLE, *n* (%)	**19 (52.78)**	**32 (18.29)**	**4.99 (2.34–10.66)**	**＜0.001**
GC, *n* (%)				
No	7 (19.44)	78 (44.57)	1.00	
GC ≥ 20 mg/day	**26 (72.22)**	**66 (37.71)**	**4.39 (1.79–10.76)**	**0.001**
GC＜ 20 mg/day	3 (8.33)	31 (17.71)	1.08 (0.26–4.44)	1.000
Immunosuppressants, *n* (%)				
No	4 (11.11)	34 (19.43)	1.00	
Yes	32 (88.89)	141 (80.57)	1.93 (0.64–5.82)	0.244
Anti‐TNF therapy, *n* (%)				
No	34 (94.44)	141 (80.57)	1.00	
Yes	2 (5.56)	34 (19.43)	0.24 (0.56–1.07)	0.061
Anti‐TB therapy, *n* (%)				
No	33 (91.67)	151 (86.29)	1.00	
Yes	3 (8.33)	24 (13.71)	0.57 (0.16–2.01)	0.384
TB history, *n* (%)				
No	27 (75.00)	164 (93.71)	1.00	
Yes	**9 (25.00)**	**11 (6.29)**	**4.97 (1.88–13.12)**	**0.001**

*Note:* Variables with boldfaced OR values in this table indicate statistically significant differences in the univariate logistic regression analysis (*p* < 0.05). Abbreviations: ATB, active tuberculosis; GC, glucocorticoid; LTBI, latent tuberculosis infection; OR, odds ratio; SLE, systemic lupus erythematosus; TB, tuberculosis.

**Table 4:** Logistic regression analysis of risk factors for progression of LTBI to ATB.

Variables	*B*	SE	Wals	OR (95% CI)	*p* value
SLE	0.389	0.529	0.542	1.48 (0.52–4.16)	0.462
GC ≥ 20 mg/day	1.279	0.537	5.685	3.59 (1.26–10.29)	0.017
TB history	0.541	0.591	0.838	1.72 (0.54–5.48)	0.360

Abbreviations: GC, glucocorticoid; LTBI, latent tuberculosis infection; OR, odds ratio; SLE, systemic lupus erythematosus; TB, active tuberculosis; TB, tuberculosis.

## 4 Discussion

Currently, there is a lack of comprehensive epidemiological data regarding the occurrence of LTBI among individuals with rheumatic conditions in China. And using IGRA as a discriminatory diagnostic tool to differentiate between ATB and LTBI, it has been estimated that the prevalence of LTBI in China′s young population aged twenties ranges from 14% to 27% [24, 25]. Research conducted in Brazil, China, South Africa, and other countries with a high TB burden has demonstrated that the occurrence of LTBI in individuals with rheumatic conditions ranges from 12.9% to 29.5% [5, 26, 27]. These findings are consistent with our own findings that this population has an incidence of 25.33%, yet only 18.60% are screened for LTBI. Increasing the screening rate for LTBI may reduce the incidence. Among the various immunosuppressants, high‐dose GC is considered the primary pharmacological agent for managing RD. Due to research limitations, we retrospectively analyzed the incidence of progression from LTBI to ATB among inpatients who had undergone radiological examinations. We found that the incidence of ATB was 14.57%, which was significantly higher than that of the general population. These findings indicate the necessity of performing radiological examinations and exploring the risk factors for the progression to ATB in RD patients with LTBI. Our findings indicate that the administration of GC at a dosage of 20 mg/day or higher is an independent risk factor for the progression to ATB in rheumatic conditions with LTBI (OR = 3.59, 95% CI: 1.26–10.29, *p* = 0.017). This observation aligns with previous research studies, thereby reinforcing the consistency of our results. Previous research has demonstrated that the administration of high doses of GC constitutes a significant risk factor for TB [28, 29]. This is primarily attributed to the ability of GC to inhibit T cell activation [30], resulting in diminished proliferative response and cytokine production, as well as the induction of lymphocyte redistribution from the bloodstream, resulting in peripheral lymphocytopenia and reduced host immunity.

Numerous studies have consistently demonstrated that patients undergoing treatment with anti‐TNF biologics face a potential risk of activating LTBI [31, 32, 33]. It is established that TNF plays a pivotal role in the host′s response to infection. Given its impact on the transportation of cells to the site of infection, it facilitates the formation of granulomas that effectively restrict disease progression, enhances the phagocytic capability of macrophages, and promotes the elimination of viable intracellular bacteria. Furthermore, TNF plays a crucial role in preserving the structural stability of the granuloma. Consequently, the administration of TNF antagonists could potentially trigger the LTBI reactivation [34, 35]. Nevertheless, our research findings indicate that TNF antagonist usage is not a contributing factor in the activation of LTBI in individuals with rheumatic conditions. Instead, this result may be attributed to the patients′ inherent immune deficiency or the effects of anti‐TB treatment. This discovery aligns with a previous study, which demonstrated that patients who had completed appropriate anti‐TB therapy before commencing anti‐TNF therapy did not exhibit an elevated risk of developing TB [36, 37]. In four observational studies, it was found that patients with LTBI who underwent preventive treatment experienced a 65% decrease in the risk of developing tuberculosis compared to those who declined treatment (relative risk [RR] 0.35, 95% CI: 0.15–0.82) [14, 38]. These findings suggest that appropriate preventive anti‐TB management can significantly reduce the incidence of ATB. Our study revealed that 20.59% of patients received prophylactic anti‐TB therapy before initiating anti‐TNF therapy. Numerous studies strongly advocate for TB screening before rheumatic therapy [39].

This study represents the initial exploration into the determinants influencing the progression from LTBI to ATB in rheumatic patients. The enrolled population underwent a meticulous screening process to exclude individuals with clinically indeterminate ATB. However, the study was limited by the fact that it could only review information from the medical records of hospitalized patients. Firstly, while we were able to report the incidence of rheumatic patients with LTBI, we were unable to provide data on its prevalence in the general population. Secondly, it is important to note that this study classified T‐SPOT.TB‐positive patients as LTBI patients, which may introduce false‐positive background interference. Finally, based on the imaging examinations, we preliminarily explored the factors influencing the progression of patients with RD from LTBI to ATB. However, we did not stratify the degree of disease progression in these patients, and the sample size was small. These two factors may affect the analysis of risk factors.

## 5 Conclusions

The findings of our study suggest that the incidence of progression from LTBI to ATB among Chinese individuals with RD is substantially higher than that in the general population. Taken together, these findings highlight the significant deficiencies in the current screening protocols and drug management for rheumatic patients with latent tuberculosis infection in clinical practice. Therefore, there is an urgent need to establish expert consensus to optimize the diagnostic and treatment processes for rheumatic patients, so as to improve the overall quality of medical services for this patient population. This is mainly because rheumatologists in China often overlook the imaging examination of patients with LTBI. Moreover, GC therapy is extensively utilized in clinical practice, and our study also reveals that medium‐ and high‐dose GC serve as independent risk factors for LTBI activation. These discoveries collectively underscore the significant deficiencies in the screening protocols and drug management for RD patients with LTBI in current clinical practice. Consequently, there is an urgent necessity to establish an expert consensus to optimize the diagnosis and treatment process of RD, aiming to enhance the overall quality of medical care for this patient population.

## Author Contributions

Xiaoyan Hao collected the data, Lei Zhou analyzed chest CT/X‐ray mention radiograph, Fengjuan Wang and Jiayun Liu designed the study, analyzed the data, and wrote the final manuscript.

## Ethics Statement

The study protocol was approved by the institutional ethics committee of the First Affiliated Hospital of the Fourth Military Medical University (reference number KY20252063‐F‐2). Our analysis focused on laboratory‐based clinical data that adhered to the standards outlined in the Helsinki Declaration. The requirement for informed consentF was waived by the First Affiliated Hospital of Fourth Military Medical University.

## Conflicts of Interest

The authors declare no conflicts of interest.

## Data Availability

The datasets utilized and/or examined in the present study can be obtained from the corresponding author upon a reasonable request. Upon reasonable request, the corresponding author will provide the datasets used and/or analyzed in this study.

## References

[1] L. Goldman and A. I. Schafer , Goldman‐Cecil Medicine (Elsevier, 2019).

[2] U. Mack , G. B. Migliori , M. Sester , et al., “LTBI: Latent Tuberculosis Infection or Lasting Immune Responses to *M. tuberculosis*? A TBNET Consensus Statement,” European Respiratory Journal 33 (2009): 956–973, 10.1183/09031936.00120908. 19407047

[3] W. H. Organization , Global Tuberculosis Report 2023 (World Health Organization, 2023).

[4] Z. Li , “A New Look at Rheumatology in China—Opportunities and Challenges,” Nature Reviews Rheumatology 11 (2015): 313–317, 10.1038/nrrheum.2014.218. 25599919

[5] C. Pettipher and R. Benitha , “Tuberculosis in Biologic Users for Rheumatic Diseases: Results From the South African Biologics Registry (SABIO),” Annals of the Rheumatic Diseases 79 (2020): 292–299, 10.1136/annrheumdis-2019-216128. 31791950

[6] S. Chandrashekara , R. Panchagnula , and Y. Chennupati , “Prevalence of LTBI in Patients With Autoimmune Diseases and Accuracy of IGRA in Predicting TB Relapse,” Rheumatology 62 (2023): 3952–3956, 10.1093/rheumatology/kead315. 37348542

[7] S. M. Jamil , E. Oren , G. W. Garrison , et al., “Diagnosis of Tuberculosis in Adults and Children,” Annals of the American Thoracic Society 14 (2017): 275–278, 10.1513/AnnalsATS.201608-636CME. 28146376

[8] C. Primaturia , L. Reniarti , and H. Nataprawira , “Comparison Between the Interferon γ Release Assay‐QuantiFERON Gold Plus (QFT‐Plus)‐and Tuberculin Skin Test (TST) in the Detection of Tuberculosis Infection in Immunocompromised Children,” Pulmonary Medicine 2020 (2020): 7159485, 10.1155/2020/7159485. 32455014 PMC7238328

[9] D. Caillet Portillo , X. Puéchal , M. Masson , et al., “Diagnosis and Treatment of Tropheryma Whipplei Infection in Patients With Inflammatory Rheumatic Disease: Data From the French Tw‐IRD Registry,” Journal of Infection 88 (2024): 132–138, 10.1016/j.jinf.2023.12.010. 38141787

[10] S. K. Brode , F. B. Jamieson , R. Ng , et al., “Increased Risk of Mycobacterial Infections Associated With Anti‐Rheumatic Medications,” Thorax 70 (2015): 677–682, 10.1136/thoraxjnl-2014-206470. 25911222

[11] P. Brassard , A. M. Lowe , S. Bernatsky , A. Kezouh , and S. Suissa , “Rheumatoid Arthritis, Its Treatments, and the Risk of Tuberculosis in Quebec, Canada,” Arthritis Care & Research 61 (2009): 300–304, 10.1002/art.24476. 19248128

[12] C. Herzmann , G. Sotgiu , O. Bellinger , et al., “Risk for Latent and Active Tuberculosis in Germany,” Infection 45 (2017): 283–290, 10.1007/s15010-016-0963-2. 27866367 PMC5488071

[13] W. Long , F. Cai , X. Wang , N. Zheng , and R. Wu , “High Risk of Activation of Latent Tuberculosis Infection in Rheumatic Disease Patients,” Infectious Diseases 52 (2020): 80–86, 10.1080/23744235.2019.1682187. 31656117

[14] X. Liu , L. Zhang , F. Zhang , et al., “Prevalence and Risk Factors of Active Tuberculosis in Patients With Rheumatic Diseases: A Multi‐Center, Cross‐Sectional Study in China,” Emerging Microbes & Infections 10 (2021): 2303–2312, 10.1080/22221751.2021.2004864. 34753408 PMC8654396

[15] O. Immunotec , T‐SPOT.TB Package Insert PI‐TB‐IVD‐UK‐v3 (Oxford Immunotec Ltd, 2016).

[16] M. Pai , A. Menzies , and D. Zwerling , “Systematic Review: T‐Cell–Based Assays for the Diagnosis of Latent Tuberculosis Infection: An Update,” Annals of Internal Medicine 149 (2008): 177–184. 18593687 10.7326/0003-4819-149-3-200808050-00241 PMC2951987

[17] Y. A. Kang , H. W. Lee , S. S. Hwang , et al., “Usefulness of Whole‐Blood Interferon‐γ Assay and Interferon‐γ Enzyme‐Linked Immunospot Assay in the Diagnosis of Active Pulmonary Tuberculosis,” Chest 132 (2007): 959–965, 10.1378/chest.06-2805. 17505029

[18] A. E. Boyd , A. Ashcroft , M. Lipman , and G. H. Bothamley , “Limited Added Value of T‐SPOT.TB Blood Test in Diagnosing Active TB: A Prospective Bayesian Analysis,” Journal of Infection 62 (2011): 456–461, 10.1016/j.jinf.2011.04.003. 21570124 PMC3116095

[19] Y. Li , L. Zhang , X. Liu , et al., “The Role of In Vitro Interferonγ‐Release Assay in Differentiating Intestinal Tuberculosis From Crohn′S Disease in China,” Journal of Crohn′s and Colitis 6 (2012): 317–323, 10.1016/j.crohns.2011.09.002. 22405168

[20] X. H. Cheng , S. N. Bian , Y. Q. Zhang , et al., “Diagnostic Value of T‐Cell Interferon‐γ Release Assays on Synovial Fluid for Articular Tuberculosis: A Pilot Study,” Chinese Medical Journal 129 (2016): 1171–1178, 10.4103/0366-6999.181958. 27174325 PMC4878162

[21] A. C. Pettit , J. E. Stout , R. Belknap , et al., “Optimal Testing Choice and Diagnostic Strategies for Latent Tuberculosis Infection Among US‐Born People Living With Human Immunodeficiency Virus (HIV),” Clinical Infectious Diseases 73 (2021): e2278, 10.1093/cid/ciaa1135. 32761083 PMC8678585

[22] S. S. Jick , E. S. Lieberman , M. U. Rahman , and H. K. Choi , “Glucocorticoid Use, Other Associated Factors, and the Risk of Tuberculosis,” Arthritis Care & Research 55 (2006): 19–26, 10.1002/art.21705. 16463407

[23] J. A. Singh , D. E. Furst , A. Bharat , et al., “2012 Update of the 2008 American College of Rheumatology Recommendations for the Use of Disease‐Modifying Antirheumatic Drugs and Biologic Agents in the Treatment of Rheumatoid Arthritis,” Arthritis Care & Research 64 (2012): 625–639, 10.1002/acr.21641. 22473917 PMC4081542

[24] J. Zhao , Y. Wang , H. Wang , et al., “Low Agreement Between the T‐SPOT.TB Assay and the Tuberculin Skin Test Among College Students in China,” International Journal of Tuberculosis and Lung Disease 15 (2011): 134. PMC3118013 21276310

[25] X. Wu , Y. Hou , Y. Liang , et al., “Evaluation of a Tuberculosis Whole‐Blood Interferon‐γ Chemiluminescent Immunoassay Among Chinese Military Recruits,” Molecular Diagnosis & Therapy 15 (2011): 341–346, 10.1007/bf03256469. 22149482

[26] M. Fornaro , S. Stano , D. Goletti , et al., “Prevalence and Management of Tuberculosis Infection in Apulian Rheumatologic Patients Treated With Biologics: An Observational Cohort 10‐Year Study From the BIOPURE Registry,” European Journal of Clinical Investigation 53 (2023): e13913, 10.1111/eci.13913. 36435984

[27] L. Gao , W. Lu , L. Bai , et al., “Latent Tuberculosis Infection in Rural China: Baseline Results of a Population‐Based, Multicentre, Prospective Cohort Study,” Lancet Infectious Diseases 15 (2015): 310–319, 10.1016/s1473-3099(14)71085-0. 25681063

[28] S. W. Lai , C. L. Lin , and K. F. Liao , “Nation‐Based Case‐Control Study Investigating the Relationship Between Oral Corticosteroids Use and Pulmonary Tuberculosis,” European Journal of Internal Medicine 43 (2017): 53–57, 10.1016/j.ejim.2017.05.020. 28554781

[29] R. Gopalaswamy and S. Subbian , “Corticosteroids for COVID‐19 Therapy: Potential Implications on Tuberculosis,” International Journal of Molecular Sciences 22 (2021): 3773, 10.3390/ijms22073773. 33917321 PMC8038708

[30] G. E. Fragoulis , E. Nikiphorou , M. Dey , et al., “2022 EULAR Recommendations for Screening and Prophylaxis of Chronic and Opportunistic Infections in Adults With Autoimmune Inflammatory Rheumatic Diseases,” Annals of the Rheumatic Diseases 82 (2023): 742–753, 10.1136/ard-2022-223335. 36328476

[31] C. Anton , F. D. Machado , J. M. A. Ramirez , et al., “Latent Tuberculosis Infection in Patients With Rheumatic Diseases,” Jornal Brasileiro de Pneumologia 45 (2019): e20190023, 10.1590/1806-3713/e20190023. 31038654 PMC6733747

[32] V. L. Dias and K. M. Storrer , “Prevalence of Latent Tuberculosis Infection Among Patients With Interstitial Lung Disease Requiring Immunosuppression,” Jornal brasileiro de pneumologia: publicacao oficial da Sociedade Brasileira de Pneumologia e Tisilogia 48 (2022): 20210382, 10.36416/1806-3756/e20210382. PMC8963748 35352793

[33] R. Scrivo and O. Armignacco , “Tuberculosis Risk and Anti‐Tumour Necrosis Factor Agents in Rheumatoid Arthritis: A Critical Appraisal of National Registry Data,” International Journal of Rheumatic Diseases 17 (2014): 716–724, 10.1111/1756-185X.12375. 24725559

[34] C. L. Yonekura , R. D. R. Oliveira , D. C. Titton , et al., “Incidência de tuberculose em pacientes com artrite reumatoide em uso de bloqueadores do TNF no Brasil: dados do Registro Brasileiro de Monitoração de Terapias Biológicas BiobadaBrasil,” Revista brasileira de reumatologia 57, no. Suppl 2 (2017): 477–483, 10.1016/j.rbre.2017.05.005. 28739353

[35] N. Jahnich and P. D. Arkwright , “Regional Risk of Tuberculosis and Viral Hepatitis With Tumor Necrosis Factor‐Alpha Inhibitor Treatment: A Systematic Review,” Frontiers in Pharmacology 14 (2023): 1046306, 10.3389/fphar.2023.1046306. 36744250 PMC9894886

[36] I. Solovic , M. Sester , J. J. Gomez‐Reino , et al., “The Risk of Tuberculosis Related to Tumour Necrosis Factor Antagonist Therapies: A TBNET Consensus Statement,” European Respiratory Journal 36 (2010): 1185–1206, 10.1183/09031936.00028510. 20530046

[37] F. Cantini , C. Nannini , L. Niccoli , et al., “Guidance for the Management of Patients With Latent Tuberculosis Infection Requiring Biologic Therapy in Rheumatology and Dermatology Clinical Practice,” Autoimmunity Reviews 14 (2015): 503–509, 10.1016/j.autrev.2015.01.011. 25617816

[38] J. W. Ai , S. Zhang , Q. L. Ruan , et al., “The Risk of Tuberculosis in Patients With Rheumatoid Arthritis Treated With Tumor Necrosis Factor‐α Antagonist: A Metaanalysis of Both Randomized Controlled Trials and Registry/Cohort Studies,” Journal of Rheumatology 42 (2015): 2229–2237, 10.3899/jrheum.150057. 26472414

[39] G. Evangelatos , V. Koulouri , A. Iliopoulos , and G. E. Fragoulis , “Tuberculosis and Targeted Synthetic or Biologic DMARDs, Beyond Tumor Necrosis Factor Inhibitors,” Therapeutic Advances in Musculoskeletal Disease 12 (2020): 1759720X20930116, 10.1177/1759720X20930116. PMC7309385 32612710

